# Structural Basis of Chemokine CXCL8 Monomer and Dimer Binding to Chondroitin Sulfate: Insights into Specificity and Plasticity

**DOI:** 10.3390/biom16010124

**Published:** 2026-01-12

**Authors:** Bryon P. Mahler, Balaji Nagarajan, Nehru Viji Sankaranarayanan, Prem Raj B. Joseph, Umesh R. Desai, Krishna Rajarathnam

**Affiliations:** 1 Department of Biochemistry and Molecular Biology, University of Texas Medical Branch, Galveston, TX 77555, USA; bmahler@iu.edu (B.P.M.); premrajb.joseph@gmail.com (P.R.B.J.); 2 Sealy Center for Structural Biology and Molecular Biophysics, University of Texas Medical Branch, Galveston, TX 77555, USA; 3 Center for Drug Discovery, Department of Medicinal Chemistry, Virginia Commonwealth University, Richmond, VA 23219, USA; bnagarajan@vcu.edu (B.N.); nvsankaranar@vcu.edu (N.V.S.); urdesai@vcu.edu (U.R.D.); 4 Center for Biomarker Research & Precision Medicine, Virginia Commonwealth University, Richmond, VA 23219, USA; 5 Department of Microbiology and Immunology, University of Texas Medical Branch, Galveston, TX 77555, USA; 6 Institute for Human Infections and Immunity, University of Texas Medical Branch, Galveston, TX 77555, USA

**Keywords:** chemokine, CXCL8, interleukin-8, glycosaminoglycan, chondroitin sulfate, heparan sulfate, heparin, nuclear magnetic resonance, molecular dynamics, proteoglycan, neutrophil

## Abstract

Chemokines play a central role in orchestrating neutrophil recruitment from the bloodstream and determining their effector functions at sites of infection. Chemokine activity is determined by three key properties: reversible monomer–dimer equilibrium, binding to glycosaminoglycans (GAGs), and signaling through the GPCR class of receptors CXCR1 and CXCR2. In this study, we investigated the structural basis of CXCL8 monomer and dimer binding to GAG chondroitin sulfate (CS) using nuclear magnetic resonance (NMR) spectroscopy, docking, and molecular dynamics (MD) measurements. Our studies reveal that both the monomer and dimer use essentially the same set of basic residues for binding, that the interface is extensive, that the dimer is the high-affinity CS ligand, and that the CS-binding residues form a contiguous surface within a monomer. Several of these residues also participate in receptor interactions, suggesting that CS-bound CXCL8 is likely impaired in its ability to bind receptors. Notably, we observe that the same basic residues are involved in binding CS and heparin/heparan sulfate, even though these GAGs differ in backbone structures and sulfation patterns. We conclude that the strategic distribution and topology of basic residues on the CXCL8 scaffold enable engagement with diverse GAG structures, which likely allows fine-tuning receptor signaling to regulate neutrophil trafficking and effector functions.

## 1. Introduction

A hallmark of the host immune response is the recruitment of circulating neutrophils to sites of infection [1,2]. Chemokines released by resident cells play a crucial role in recruiting neutrophils from the bloodstream to the affected tissues. Three characteristic properties of chemokines govern neutrophil migration and function: reversible existence as monomers and dimers, binding to glycosaminoglycans (GAGs) such as heparan sulfate (HS) and chondroitin sulfate (CS), and signaling through the GPCR class receptors CXCR1 and CXCR2 [3,4,5,6,7,8,9,10,11,12,13,14,15,16,17,18]. As extracellular proteins, chemokines interact with GAGs throughout their journey toward the vasculature, from crossing epithelial and endothelial barriers to traversing the extracellular matrix (ECM), and GAG-bound chemokines ultimately guide neutrophils from the blood to the destination site.

HS and CS are linear sulfated polysaccharides that are covalently linked to core proteins known as proteoglycans (PGs) [19,20,21,22]. Syndecans (Sdc), a class of PGs, play a central role in chemokine-mediated neutrophil trafficking. For instance, knockout of syndecan-1 (Sdc-1) or syndecan-4 (Sdc-4), which are expressed on endothelial and epithelial cells, leads to increased susceptibility to infection and impaired neutrophil recruitment in mice [22,23,24]. Whereas Sdc-1 carries both HS and CS chains, Sdc-4 carries only HS chains. In the ECM, chemokines interact with PGs such as perlecan, versican, and decorin, which carry varying amounts of CS and HS. GAG interactions determine the relative levels of free and bound monomer and dimer, receptor interactions, and the formation and maintenance of chemokine gradients. These observations collectively indicate that binding to both CS and HS is crucial for effective neutrophil responses.

Humans express seven chemokines, characterized by the N-terminal ‘ELR’ motif, that recruit neutrophils: CXCL1, CXCL2, CXCL3, CXCL5, CXCL6, CXCL7, and CXCL8 [25,26]. These chemokines dimerize at micromolar (µM) concentrations, with the actual dimerization constant varying (~1 to 100 µM) among the different members and also for a given member due to solution conditions such as pH and ionic strength [15,27,28,29,30,31]. During neutrophil recruitment, spatial and temporal changes in chemokine concentration and GAG interactions result in varying levels of monomers and dimers in the free and bound firms. Further, the receptor signaling activities of the monomer and dimer, both in their free and GAG bound forms, differ due to the structural and dynamic characteristics of the chemokine between the two states.

The basic building blocks of CS are repeating disaccharide units of D-glucuronic acid (GlcA) and *N*-acetyl-D-galactosamine (GalNAc). The majority of CS disaccharides carry one sulfate group, either at the 4- or 6- position of GalNAc. The basic building blocks of HS are repeating disaccharide units of D-glucuronic acid (GlcA) and *N*-acetyl-D-glucosamine (GlcNAc). On average, each HS disaccharide carries two or more sulfate groups. In addition to *N*-sulfation, GlcNAc can undergo 6-*O*-sulfation, while GlcA can undergo 2-*O*-sulfation. Therefore, understanding how differences in GAG structure and sulfation pattern and differences between chemokine monomer and dimer structures determine binding is essential for delineating the molecular mechanisms underlying chemokine-mediated neutrophil responses.

CXCL8 is one of the best-studied chemokines and has served as a model protein for characterizing GAG interactions since its discovery [32,33,34,35,36,37,38]. Structures of the wild-type (WT) dimer and a trapped monomer have been determined [39,40,41]. Administering engineered CXCL8 monomer and dimer variants, as well as the WT protein, to naïve uninfected mice has shown that they have differential in vivo neutrophil recruitment activities. The monomer, despite having higher levels of receptor activity, can have lower neutrophil recruitment activity, and the dimer, despite having less receptor activity, can have more neutrophil recruitment activity, indicating that GAG interactions play an important role in determining recruitment activity [42,43]. Here, we investigated the structural basis of CXCL8 monomer and dimer binding to CS using nuclear magnetic resonance (NMR) spectroscopy, combined with experimental data-driven molecular docking and molecular dynamics (MD) simulations. NMR data show that the same set of basic residues mediate binding for both monomer and dimer, the binding interface is extensive, the dimer is the high-affinity CS ligand, and the CS binding residues form a contiguous surface within a monomer. Moreover, several of these residues are also involved in receptor interactions, indicating that CS-bound CXCL8 is likely impaired in receptor binding.

We had previously characterized the binding of heparin oligosaccharides to CXCL8 as a model for HS interactions [18,44]. In this study, we have now characterized the binding of HS polymer to CXCL8 using NMR spectroscopy, and show that the residues mediating binding to heparin and HS are essentially identical. Importantly, essentially the same residues are also involved in CS interactions. Despite the 2-fold lower sulfation density in CS compared to HS, the observation that CXCL8 binds CS and HS/heparin in a similar manner is striking. Further, CXCL8–CS interactions differ markedly from those of related chemokines CXCL1 and CXCL5 [16]. We conclude that the strategic distribution and topology of basic residues, both those shared with other chemokines and those unique to CXCL8, enable engagement with diverse GAG structures, thereby fine-tuning receptor signaling and regulating neutrophil trafficking and effector functions.

## 2. Materials and Methods

### 2.1. NMR Experiments

NMR spectra were recorded at 30 °C using Bruker Avance III 600 and 800 MHz spectrometers equipped with QCI and TCI cryogenic probes (Bruker BioSpin, Rheinstetten, Germany). Titration of CS oligosaccharides to ^15^N-labeled CXCL8 was carried out in 50 mM sodium phosphate buffer containing 1 mM sodium azide, 1 mM 2,2-dimethyl-2silapentanesulfonic acid (DSS) and 10% (*v*/*v*) ^2^H_2_O. The pH of the buffer ranged from 5.5 to 7.5 depending on the experiment and is stated along with the results. The expression and purification of ^15^N-CXCL8 WT and V27P/E29P monomer variants were carried out as described [45]. The chemical shift perturbations (CSP, Δδ_cal_) are a weighted Euclidean distance denoted by the individual change in proton (Δδ_H_) and nitrogen (Δδ_N_) chemical shifts. Apparent dissociation constant (K_d_) was determined by fitting the binding-induced chemical shift changes as described [46]. CS oligosaccharides were purchased from Iduron (Manchester, UK). According to the manufacturer, the oligosaccharides were purified using high-resolution gel filtration chromatography of partial chondroitin ABC lyase digestion of CS from shark cartilage, and the GalNAc unit of the disaccharide was sulfated either at C4 (labeled as CS4S) or at C6 (CS6S). All GAGs contained uronic acid at the non-reducing end and a C4-C5 double bond as a result of the endolytic action of the chondroitin ABC lyase.

### 2.2. Docking of CXCL8-CS Complexes

A virtual library of CS4S and CS6S octasaccharides was generated using an in-house glycan builder. Disaccharide units of [GlcA–GalNAc(SO_3_^−^)] were constructed with sulfation either at the C4 or C6 positions using UCSF Chimera, and the structures were energy-minimized using the AMBER force field [47]. Heavy-atom coordinates were constrained during minimization, which was conducted for up to 10,000 steps with a convergence threshold of 0.05 kcal·mol^−1^·Å^−1^.

Molecular docking using the combinatorial virtual library screening (CVLS) protocol was performed with GOLD v5.6 software [48,49,50]. A flexible-ligand, rigid-receptor model was adopted. Binding regions on CXCL8 were defined based on NMR chemical shift perturbation (CSP) measurements as described above. Residues within an 18 Å radius from the centroid of each binding pocket were considered during docking. Each CS oligosaccharide was subjected to three independent docking runs with 100 genetic algorithm iterations per run. The six top-ranking poses were analyzed and the best pose based on GOLDScore was used for MD simulations.

### 2.3. Molecular Dynamics Simulations

The CXCL8-CS complexes obtained from the CVLS docking protocol that best agreed with the NMR data were used as starting structures for unrestrained MD simulations for 200 ns. The complexes were prepared using the LEaP module of the AMBER18 simulation suite [51]. Protein and ligand topologies were generated using the AMBER ff12SB force field for CXCL8 and GLYCAM_06j-1 for the octasaccharides [52]. The total charge of each system was neutralized by the addition of appropriate counterions. Each neutralized complex was solvated in a TIP3P water box [53], maintaining a minimum distance of 12 Å between the complex and the edges of the periodic box. System coordinates and corresponding topology files were generated for subsequent simulations.

Energy minimization was carried out in two phases with a non-bonded cutoff of 10 Å. In Phase 1, solute atoms and counterions were restrained with a harmonic potential (force constant = 100 kcal/mol·Å^2^). The water molecules were relaxed using 500 cycles of steepest descent followed by 2000 cycles of conjugate gradient minimization. In Phase 2, the entire system was minimized without restraints using 2500 cycles of conjugate gradient minimization. The molecular dynamics simulations were started by equilibrating the system. Equilibration was performed, with the integration time step being 2fs. At first, the temperature of the system was brought to 300 K using the Berendsen temperature coupling with time constant 2 ps; similarly, the system was brought to a constant pressure using isotropic position scaling. Equilibration was initially carried out for 1 ns of the total timescale with strong restraint of the solute and it was constantly reduced. The production run was performed in the NPT ensemble with an integration time step of 1 fs. Bonds involving hydrogen atoms were constrained in the SHAKE algorithm. The total trajectory was computed for 200 ns using the in-house GPU cluster for each complex. Equilibration and the simulation process were validated using the physical observables of the system. The simulation was recorded for a snapshot of 10 ps time interval. The variations in the total, potential and kinetic energies, temperature, and pressure were estimated as the function of the time, which further confirmed that the system obeyed the NPT ensemble.

### 2.4. MM-GBSA Binding Free Energy Calculations

The binding free energies of each CXCL8–CS complex were calculated using the MM-GBSA approach [51]. Trajectory frames recorded from the molecular dynamics (MD) simulations were extracted at equal time intervals, with a total of 4000 frames selected for the energy calculations. Single-residue energy decomposition (SRED) within the MM-GBSA framework was employed to estimate the individual contributions of residues to binding. All energy calculations were performed using default parameters with the Python-based (3.13.10) MM-GBSA module implemented in AmberTools18 [51].

## 3. Results

**Figure 1:**
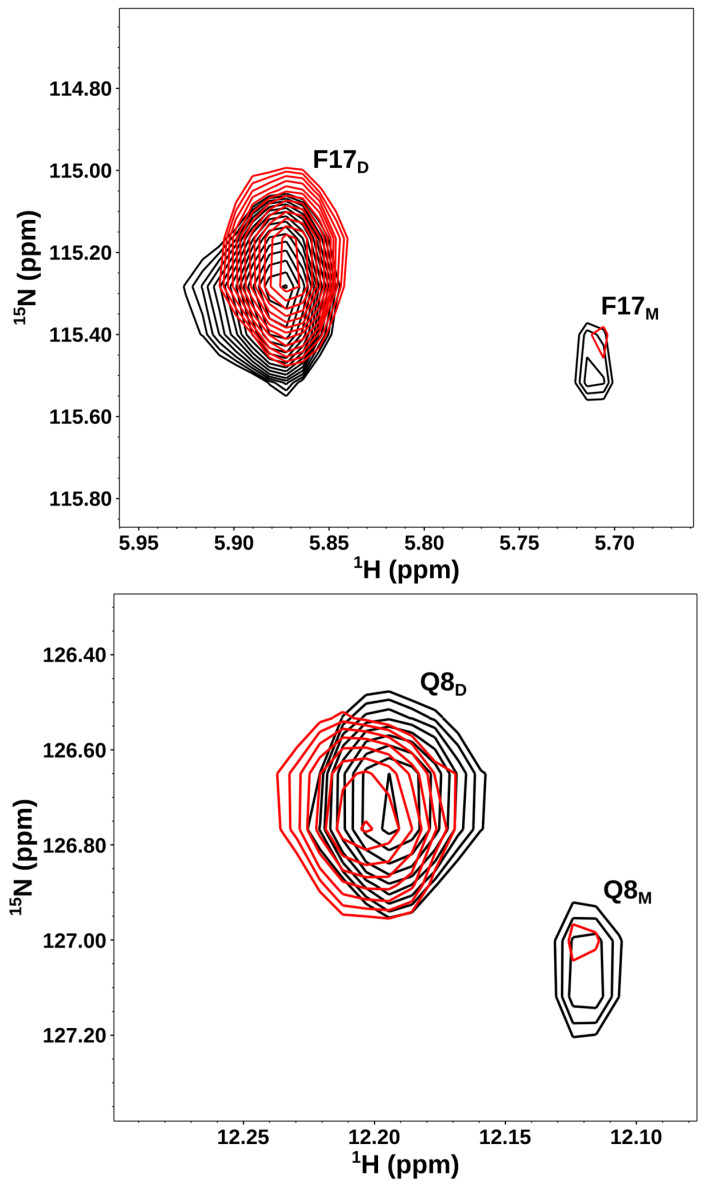
CXCL8 dimer is the high-affinity CS ligand. Sections of the ^1^H-^15^N HSQC spectra for a select set of CXCL8 residues showing the overlay in the free (black) and CS dp14-bound (red) forms. Dimer and monomer peaks are indicated by D and M, respectively. Weaker monomer and stronger dimer peaks in the bound form indicate that the dimer is the high-affinity ligand. The spectra were collected using a ~10 µM protein sample in 50 mM phosphate pH 7.5.

NMR chemical shifts are highly sensitive to binding-induced changes in the local environment. Therefore, we identified the CXCL8 residues that bind CS oligosaccharides using solution NMR spectroscopy. CXCL8 exists reversibly as a monomer and a dimer [30,54,55]. To determine the relative affinities of CS for the monomer and dimer, we characterized binding using a ~10 µM protein sample at pH 7.5. Under these conditions, distinct peaks corresponding to both the monomer and dimer could be observed in the HSQC spectrum due to slow exchange between the two forms on the NMR timescale (Figure 1). It is important to note that all four species—free monomers, free dimers, GAG-bound monomers, and GAG-bound dimers—coexist in dynamic equilibrium. On binding, the NMR signals corresponding to the monomer weaken and the dimer strengthen, indicating that the dimer is the high-affinity ligand.

We characterized the binding of CS to the CXCL8 dimer using a 50 µM protein sample at pH 6.0, where CXCL8 exists essentially as a dimer. To characterize monomer interactions, we characterized the binding of CS to the double proline (V27P/E29P) mutant using a 50 µM protein sample at pH 6.0. We have shown that this construct is monomeric and as active as the native monomer based on NMR studies and functional assays [56]. Procedures for chemical shift assignments for the WT dimer and V27P/E29P monomer have been described in our previous studies [18,44,56,57]. For both the WT dimer and the V27P/E29P monomer, CS binding-induced chemical shift changes were assigned by collecting a series of spectra on adding incremental amounts of a 5 to 10 mM CS solution to reach a final CS–protein ratio of 3:1 and 4:1 for the dimer and monomer, respectively. We observed only a single set of peaks during titration of CS to both CXCL8 monomer and dimer, indicating that free and bound forms are in the fast exchange regime on the NMR timescale.

### 3.1. CS Binding Affinities of CXCL8 Monomer and Dimer

Apparent dissociation constant (K_d_) was determined by fitting CS 14mer binding-induced chemical shift changes (Δδ_obs_). For the dimer, we calculated a K_d_ of 22 ± 8 µM, based on individual fits for seven residues that showed significant chemical shift changes (Figure S1A). A binding constant could not be determined for the monomer due to weak interactions and an absence of saturation in the binding isotherms (Figure S1B).

### 3.2. Characterization of CXCL8 Residues That Mediate CS Binding

**Figure 2:**
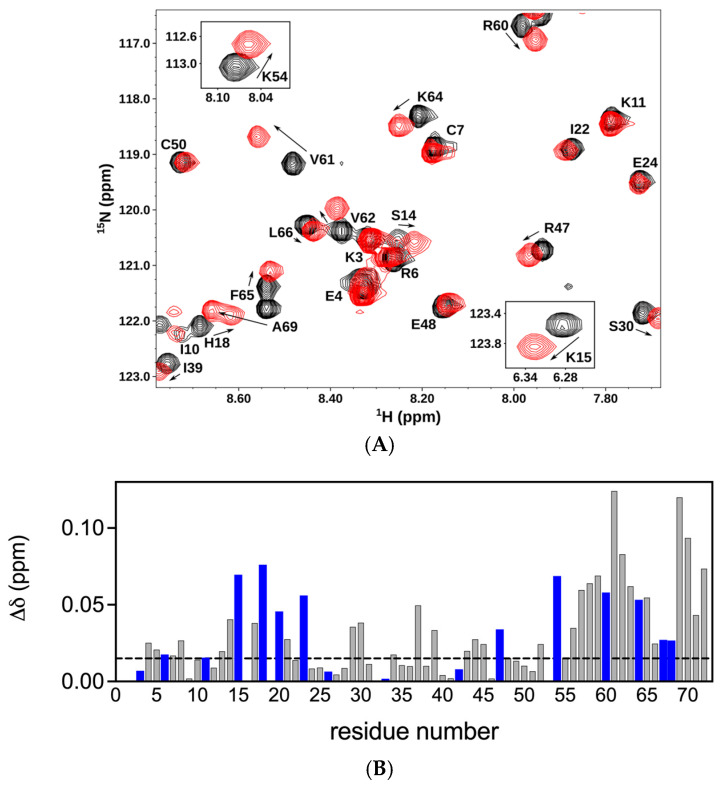
Binding of CXCL8 dimer to chondroitin sulfate. (**A**) Sections of the ^1^H-^15^N HSQC spectra showing the overlay of the CXCL8 dimer in the free (black) and CS dp14-bound (red) forms. Arrows indicate the direction of the peak movement. (**B**) Histogram plot of chemical shift changes in CXCL8 dimer on binding to CS dp14. Residues showing chemical shift perturbation (CSP) higher than the threshold (indicated by the dotted line) are implicated in binding. Basic residues lysine, arginine, and histidine are shown in blue.

We first characterized the binding of a panel of CS oligosaccharides of increasing length from an octasaccharide (dp8) to a 14mer (dp14) to the CXCL8 dimer. The chemical shift perturbation (CSP) profile was largely the same for all oligosaccharides, except that the magnitude of the shifts increased with oligosaccharide length, indicating stronger binding. Therefore, we will describe the binding interactions of the 14mer. Pronounced chemical shift changes were observed for basic residues Lys15 (K15), His18 (H18), Lys20 (K20), Lys23 (K23), Arg47 (R47), Lys54 (K54), Arg60 (R60), Lys64 (K64), Lys67 (K67), and Arg68 (R68) (Figure 2). Basic residues K3, R6, K11, R26, H33, and K42 were not perturbed. These binding residues, located in the N-loop, 40s-loop, and C-helix in the primary sequence, are clustered in the folded protein and present a positively charged surface for GAG binding. Significant chemical shift changes were also observed for acidic, polar, and non-polar buried residues that are in the proximity of the basic residues. These shift changes could be due to binding mediated by packing and H-bonding interactions, and/or could reflect structural changes and may not be due to direct binding interactions. The histogram plots of all the oligosaccharides are shown in the Supplementary Material (Figure S2).

In addition to characterizing binding from backbone chemical shift changes, we also characterized the binding from arginine side chain N**ε**H shift changes [58]. The chemical shifts in Arg N**ε** are distinctly different from those of the backbone nitrogens. We collected these data at pH 5.5, as lower pH improved spectral quality due to reduced proton exchange with the bulk solvent. CXCL8 has 5 arginines—R6, R26, R47, R60, and R68. The spectra show binding-induced chemical shift changes for R47, R60, and R68 and not for R6 and R26 (Figure S3), providing independent evidence that only the former set of residues are involved in the binding process.

**Figure 3:**
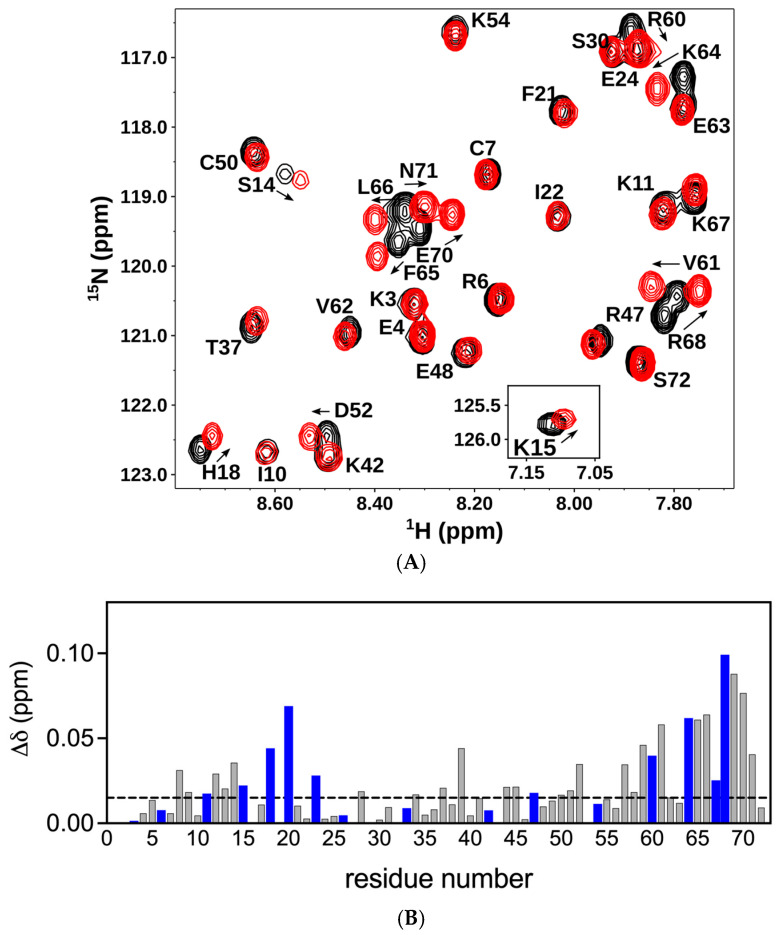
Binding of CXCL8 monomer to chondroitin sulfate. (**A**) Sections of the ^1^H-^15^N HSQC spectra showing the overlay of the CXCL8 monomer in the free (black) and CS dp14 bound (red) forms. Arrows indicate the direction of the peak movement. (**B**) Histogram plot of chemical shift changes in CXCL8 monomer on binding to CS dp14. Residues showing CSP higher than the threshold (indicated by the dotted line) are implicated in binding. Basic residues lysine, arginine, and histidine are shown in blue.

On the basis of our results for the dimer, next, we characterized the binding of CS dp14 to the CXCL8 monomer. Significant chemical shift changes were observed for residues K15, H18, K20, K23, R47, R60, K64, K67, and R68 (Figure 3A,B). These data suggest that essentially the same residues mediate binding in the monomer and the dimer. In addition, similar to the dimer, several neighboring non-basic residues in the N-loop and C-helix region show significant perturbation, suggesting that polar and packing interactions could also play a role in promoting complex formation.

**Figure 4:**
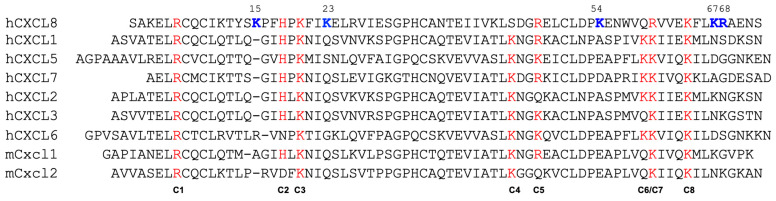
Sequences of neutrophil-activating chemokines. GAG binding residues in CXCL8 and the corresponding conserved basic residues in related chemokines are highlighted in red. Residues specific to CXCL8 are highlighted in blue and are in bold.

Previous studies of human chemokines CXCL1, CXCL5, CXCL7, and CXCL8, and mouse chemokines Cxcl1 and Cxcl2, have identified that several of the GAG-binding basic residues are conserved, while residues that are unique to a chemokine also contribute to binding. The eight conserved basic residues are highlighted in red and labeled as C1 to C8, whereas those unique to CXCL8 are shown in blue (Figure 4). Notably, not all conserved residues participate in binding in every chemokine [24,26,27,28,29], and a combination of conserved and chemokine-specific residues mediates binding in a manner that varies between chemokines and across different GAGs.

**Figure 5:**
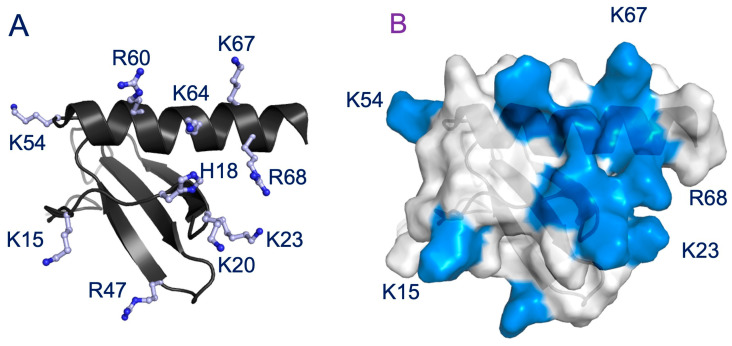
Distribution of basic residues that mediate GAG interactions. (**A**) A schematic of CXCL8 monomer showing the distribution of basic residues Arg, Lys, and His in ball and stick. (**B**) Surface plot of CXCL8 monomer with the GAG-binding residues shown in blue. Residues that are unique to CXCL8 are labeled.

NMR CSP data indicate that up to ten and nine basic residues could be involved in the binding of the dimer and monomer, respectively (Figure 2 and Figure 3). CXCL8 lacks basic residues at positions C4 and C6, but has as many as five unique GAG-binding residues (K15, K23, K54, K67, and R68). Except for K54 in the monomer, all of the unique residues show significant CSPs on CS binding. The topology and distribution of the basic residues in the CXCL8 structure suggest that no single CS binding geometry can simultaneously engage all of the residues inferred from NMR studies (Figure 5). This implies that CS may bind to multiple binding surfaces that are in dynamic equilibrium, with each surface differing slightly by including or excluding one or more residues, and/or that CSPs of some of the residues reflect indirect structural effects rather than direct binding.

### 3.3. Structural Models of CS-Bound CXCL8 Monomer and Dimer

We performed docking calculations to identify favored binding poses, followed by MD simulations to refine the complexes and gain insights into the dynamics, energetics, and pairwise interactions associated with CS4S and CS6S binding to the CXCL8 monomer and dimer. We describe residues that form direct and water-mediated intermolecular hydrogen bonds (H-bonds) and their binding free energies (ΔG). H-bonding interactions were determined using the cpptraj program [59] with a donor–acceptor distance cutoff of 3.5 Å and an angle cutoff of 45°, and binding free energies were calculated using the MM-GBSA method [60].

Consistently, the CS chains display substantial plasticity, undergoing rotational and translational motions on the highly charged protein surface. Differences in sulfation location between the CS4S and CS6S variants had minimal impact in their overall dynamic behavior in solution. The translational and rotational motions of the CS chains contributed to the interchange of H-bond interactions between the *n* (GlcA) and *n* + 1 (GalNAc(4/6)S) residues, and vice versa. Across all four simulations, the overall contribution from non-sulfate groups (carboxyl and hydroxyl) exceeded that of sulfate groups. Videos of the MD simulations of the CXCL8 monomer–CS4S and CXCL8 monomer–CS6S complexes are shown in the Supplementary Materials.

#### 3.3.1. Structural Characteristics of the Monomer–CS Complexes

**Figure 6:**
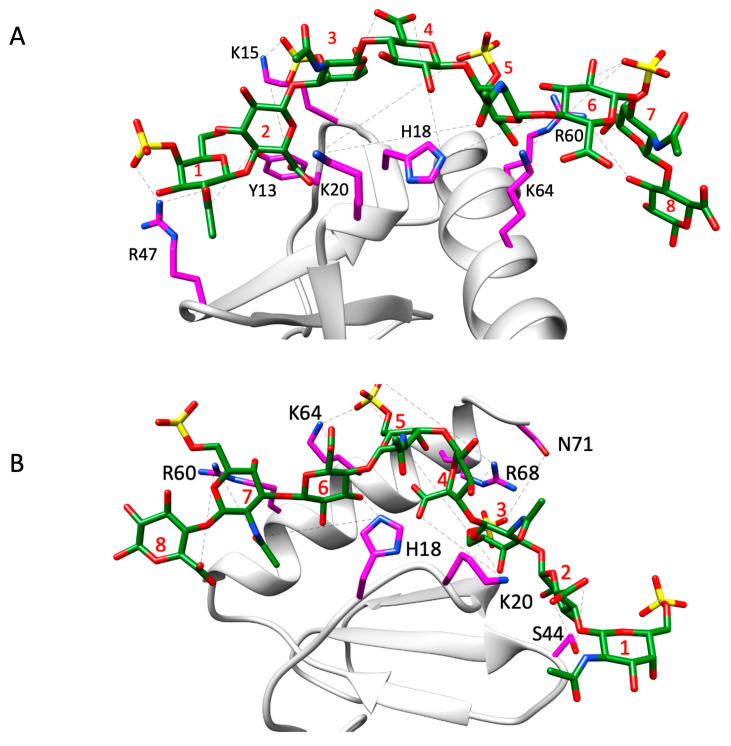
Structural models of CS4S (**A**) and CS6S (**B**) bound to the CXCL8 monomer. Interacting CXCL8 amino acid residues are displayed as magenta sticks and labeled accordingly. The CS molecule is shown as green sticks with heteroatoms color-coded. H-bonds observed across trajectory frames are indicated by black dashed lines. CS residues 1, 3, 5, and 7 correspond to GalNAc6S, while residues 2, 4, 6, and 8 correspond to GlcA. The structures shown serve as a representative frame for the full simulation trajectory.

**Figure 7:**
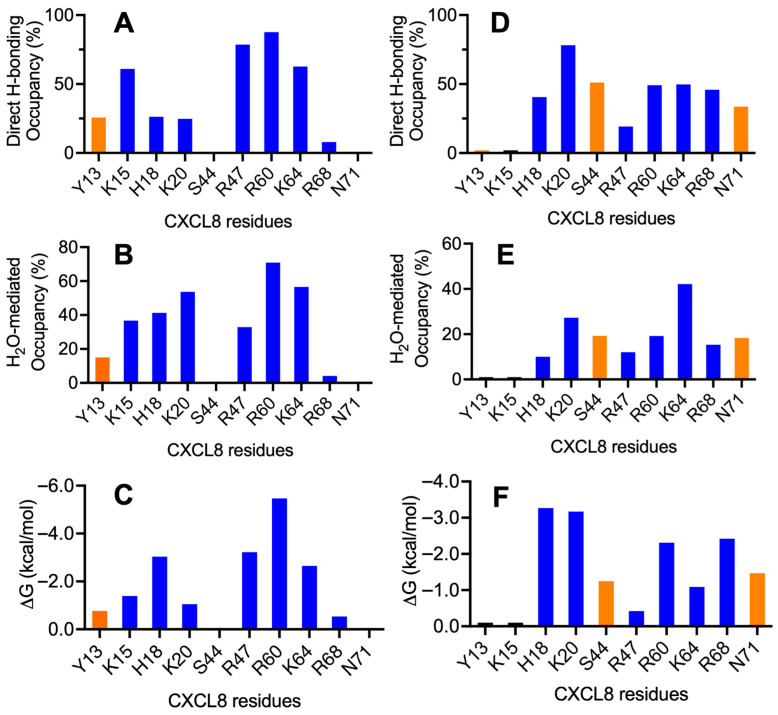
H-bonding properties and energetics of the interface residues of the CS–monomer complexes. Plots for CS4S and CS6S are shown in the left and right columns, respectively. (**A**,**D**) Occupancy of intermolecular H-bonds; (**B**,**E**) occupancy of water-mediated H-bonds; and (**C**,**F**) single-residue energy-decomposition values. Basic and polar residues are shown in blue and orange, respectively.

The favored poses consistent with the NMR data for the monomer complexes are shown in Figure 6. A summary of the H-bonding interactions and energetics of the binding-interface residues is shown in Figure 7. The structures reveal that five conserved residues—H18, K20, R47, R60, and K64—are involved in binding to both CS variants. K15 and R68, which are unique to CXCL8, are involved in binding to one CS variant or the other. In addition, polar residues Y13, S44, and N71 were also observed to interact with one CS variant or the other. Interestingly, S44 corresponds to a conserved location (C4; Figure 4) where lysine occurs in all other chemokines, suggesting that a polar residue at this position can play an equally important role in a context-dependent manner.

For the CS4S variant, the H-bond interaction at K15 arises primarily from the carboxyl group of 2-GlcA, with only a minor contribution (about 8%) from the sulfate group of 3-GalNAc4S (Figure 6). The C-helical residue R60 forms an inter-switchable interaction with the O3 position of the terminal 8-GlcA and with the sulfate group of the GalNAc4S at position 7 in the chain, although the O3 atom of 8-GlcA contributes the most.

For the CS6S variant, residue K20 plays an important role in stabilizing the complex by forming the highest number of H-bonds, contributed primarily by the carboxyl group of GlcA at position 4. In addition, the translational motion of the CS6S chain allows K20 to engage in transient interactions with the sulfate group of GalNAc6S at position 5. Although the lifetimes of these two interactions differ, each contributes nearly equally to the overall stability of the complex. The polar residue S44 interacts with the carboxyl group of GlcA at position 2, with an average H-bond distance of 2.8 Å and an angular variation of <30°. This is mainly due to the minimal variation in preferred binding of the CS6S chain. The basic residues of the C-helix also participate in translational binding from *n* to *n* + 1 residues, similar to what is observed for the CS4S chain.

#### 3.3.2. Structural Characteristics of the Dimer–CS Complexes

**Figure 8:**
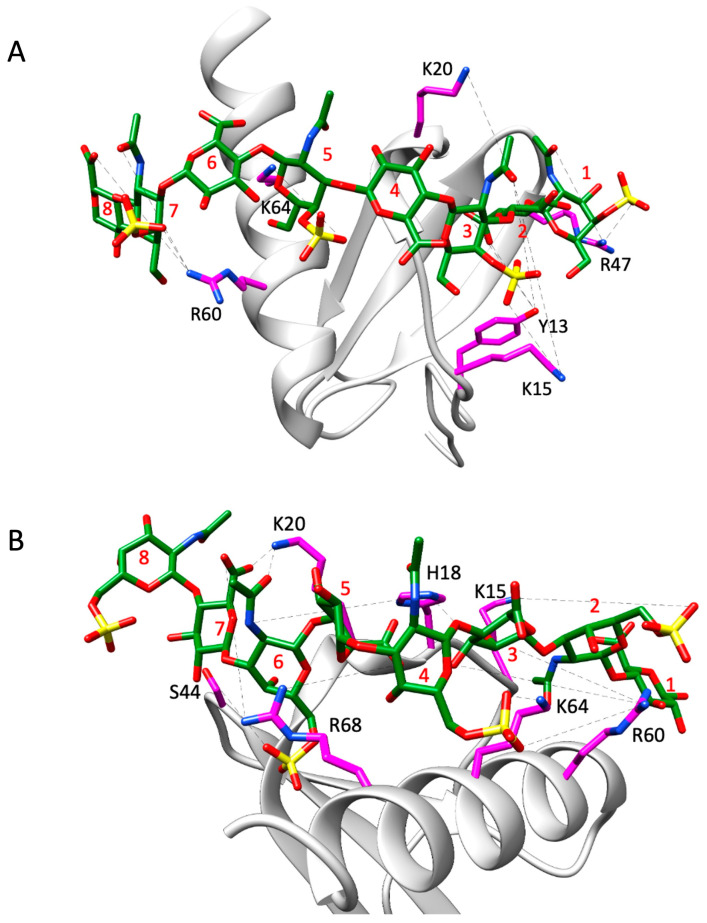
Structural models of CS4S (**A**) and CS6S (**B**) bound to a monomer in the CXCL8 dimer. Interacting CXCL8 amino acid residues are displayed as magenta sticks and labeled accordingly. The CS molecule is represented by green sticks with heteroatoms color-coded. H-bonds observed across trajectory frames are indicated by black dashed lines. CS residues 1, 3, 5, and 7 correspond to GalNAc6S, while residues 2, 4, 6, and 8 correspond to GlcA. The structures shown serve as a representative frame for the full simulation trajectory.

**Figure 9:**
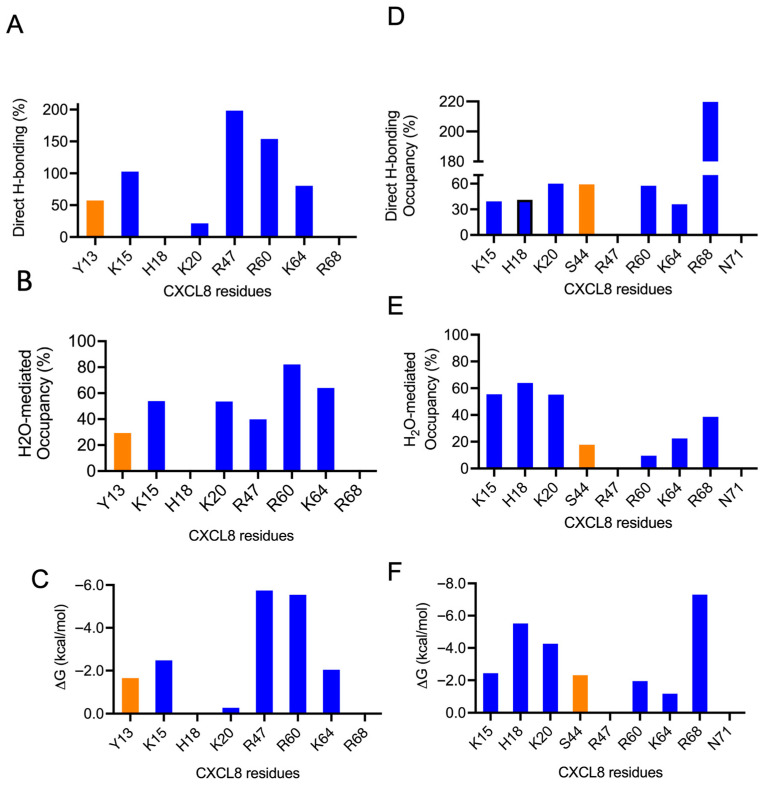
H-bonding properties and energetics of the interface residues of the CS–dimer complexes. Plots for CS4S and CS6S binding to one of the monomers of the dimer are shown in the left and right columns, respectively. (**A**,**D**) Occupancy of intermolecular H-bonds; (**B**,**E**) occupancy of water-mediated H-bonds; and (**C**,**F**) single-residue energy-decomposition values. Basic and polar residues are shown in blue and orange, respectively.

For the dimer, for both CS variants, only structural models in which binding occurred within a monomer were consistent with the experimental NMR data. Models in which GAG spanned the dimer interface were not supported by NMR data, as these poses showed interactions for C-terminal helical residues R60, K64, K67, and R68, but none for N-loop and 40s-loop residues K15, H18, K20, or R47. For both CS variants, the favored poses and interactions of basic residues with GAG sulfates and carboxylates are shown in Figure 8, and H-bonding and free energy plots are shown in Figure 9. The structural models of CS-bound dimer complexes are shown in Figure S4.

The data reveal that residues K15, K20, R60, and K64 are involved in binding to both CS variants, and Y13, H18, S44, R47, and R68 bind one or the other. Unlike the monomer, these data show that a subset of residues dominate the binding interactions. These differences are most likely due to differences in conformational dynamics between the monomer and dimer. The higher flexibility of the monomer results in interactions that are more transient, whereas the restricted motions in the dimer promote interactions that have optimal geometric and energetic complementarity and are therefore long-lived. For instance, MD data of the dimer for the CS4S complex show that the guanidinium side chain of R60 is involved in H-bonding interactions with either the 3-hydroxyl group of GlcA at position 7 or the sulfate group of GalNAc4S at position 8 throughout the trajectory, and for the CS6S complex, the guanidinium side chain of R68 is engaged in two H-bonding interactions with the carboxyl group of GlcA at position 7 throughout the trajectory.

Structural models indicate that an octasaccharide (dp8) is sufficient to cover the binding interface, and so in principle, 16 different combinations of CS variants, with either CS4S or CS6S for every disaccharide, could exist in the commercial CS that was used in the NMR titration experiments. Nevertheless, the docking and MD analyses using homogeneous CS4S and CS6S variants are able to provide structural models that are largely consistent with the NMR data. Four residues, K15, K20, R60, and K64 are involved in binding to both the CS variants, suggesting that their location and side chain properties on the CXCL8 scaffold allows them to bind both CS4S and CS6S. R47 is involved in binding only to CS4S and H18 and R68 binding only to the CS6S variant, suggesting that these residues are more restricted in their CS interactions.

Significant CSPs for K23 in both the monomer and dimer are noteworthy. This residue is unique to CXCL8, whereas the corresponding residue is polar in all other chemokines (Figure 4). Models for both the monomer and dimer show that K23 is not involved in binding to either of the CS variants, suggesting that its chemical shift changes are indirect and likely reflect local structural rearrangements caused by neighboring residues H18 and K20 engaging with CS or that the K23-bound form is a minor conformer. Similar to CS, NMR data for heparin binding also show CSPs for K23 in both the monomer and dimer, and modeling data indicate that its interactions are either transient or due to indirect structural effects [44]. The models also fail to show interactions for residues K54 and K67 to either of the CS variants in both the monomer and dimer, despite both showing significant CSPs in the NMR experiments. Both of these residues are unique to CXCL8. The models indicate that simultaneous engagement of R47 and K54 is not possible due to their relative positions in the three-dimensional structure (Figure 5). Therefore, interactions involving K54 likely represent a minor population that is not captured in our MD simulations. For K67, its CSPs may reflect indirect effects such as backbone structural rearrangements or transient interactions that were not captured in the MD studies.

### 3.4. Comparison Between Heparin/HS and CS Binding to the CXCL8 Dimer and Monomer

We previously characterized the binding of CXCL8 to heparin oligosaccharides as a model GAG for heparan sulfate [44]. Heparin was used because it is more uniformly sulfated and due to the availability of size-defined oligosaccharides that are essential for structural and modeling studies. We have now characterized the binding of CXCL8 dimer to the HS polymer and observed that the CSP profiles of HS and heparin are essentially the same. More importantly, the CSP profiles of CS and HS are also essentially the same (Figure S5). That the complete repertoire of basic residues that mediates HS binding also mediate CS binding is noteworthy, considering the differences in backbone structure and sulfation pattern, and the fact that CS is less sulfated, and on average, has only one sulfate compared to 2 to 3 in HS.

## 4. Discussion

NMR and cryo-EM studies have established that the CXCL8 N-loop residues bind to the CXCR1 and CXCR2 N-terminal domains [18,46,57,61,62,63]. Our data show that N-loop K15, H18, and K20 residues for both CXCL8 monomer and dimer are involved in CS binding. Structural models of CS-CXCL8 complexes also show these and other N-loop residues are occluded at the binding interface, suggesting that CS-bound CXCL8 will be impaired for receptor binding. Using NMR, we previously showed that CXCR1 and CXCR2 N-terminal domain peptides are unable to bind heparin-bound CXCL8 monomer or dimer complexes [18]. Further, the same subset of N-loop residues mediates GAG and receptor interactions in all human and mouse chemokines studied to date, suggesting a conserved mechanism [3,4]. However, these in vitro studies cannot definitively exclude the possibility that CS-bound CXCL8 dimer in PGs binds the receptor. In the context of in vivo PG interactions, one monomer could be bound to a single CS chain, leaving the second monomer available for receptor interactions. Experiments specifically designed to examine chemokine binding to PG GAGs at both the cell surface and within the ECM are essential to fill this knowledge gap.

The observation that the same basic residues in CXCL8 mediate binding to CS and heparin/HS differs markedly from those in CXCL1 and CXCL5. In CXCL1 and CXCL5, similar to CXCL8, N-loop, 40s-loop, and helical basic residues mediate heparin/HS binding, whereas only N-loop and 40s-loop residues mediate CS binding [15,16,64]. Further, whereas CS binds within a monomer of the dimer in CXCL1, it binds across the dimer interface in CXCL5. Notably, CXCL1 has two non-equivalent HS/heparin binding sites on opposing faces of the dimer, while the heparin/HS binding interface in CXCL5 lies within the monomer of the dimer and is distinctly different from that of CXCL8.

A previous NMR and docking study of the WT dimer reported CSPs for H18, K20, K23, K54, and K64 for both CS variants, with additional perturbations for R60, K67, and R68 observed only for CS6; however, the perturbations for K67 and R68 were minimal [38]. That study did not observe perturbations for K15 or R47 and did not discuss the perturbations observed for K23 and K54. Importantly, there are critical differences between their NMR studies and ours. Whereas they carried out NMR titrations using a 1 mM sample, we used a 50 µM sample (final protein–GAG ratio of 1:4). We could reach higher ligand excess due to our low starting protein concentration, obtain binding isotherms, and were not limited by precipitation issues. In addition, we employed a longer oligosaccharide (dp14) compared to the dp6 used in their study. These differences are expected to influence both the magnitude and the nature of the CSP profiles, thereby explaining the differences between the two studies. With respect to docking, the previous study proposed interactions of both CS variants with H18, K20, R60, K64, K67, and R68. However, those docking calculations were performed using a tetrasaccharide, whereas we employed an octasaccharide. Thus, our study comes closer to the nature of the physiological system. Overall, the nature and depth of our investigation, including the use of a physiologically relevant longer oligosaccharide and detailed residue-specific interaction analysis, makes it difficult to perform direct, head-to-head comparison between the two studies. A recent computational docking study reported that the CXCL8 monomer binds CS with higher affinity than the dimer [65]. These observations contradict our NMR studies, which are unambiguous in showing that the dimer is the high-affinity CS ligand. Further, our previous NMR studies of four human and two mouse chemokines binding to heparin, HS, and CS have consistently shown that the dimer is the high-affinity ligand. These results highlight both the importance of experimental validation and the limitations of docking analyses.

## 5. Conclusions

Our current studies, together with those reported previously for CXCL8 and related proteins and their binding to GAGs with different backbone structures and sulfation patterns, provide several important insights—namely, that their GAG interactions are not promiscuous but highly specific. Although these chemokines share common characteristics, such as dimers serving as high-affinity GAG ligands and a dynamic binding interface, their binding interactions, including geometries, are rich and diverse. Using a combination of conserved and unique basic residues as a platform, the specific interactions and plasticity of the binding interface allow each chemokine to bind different GAGs in ways that allow fine regulation of their in vivo receptor activation and neutrophil effector functions. Therefore, the impact of GAG interactions on monomer and dimer populations and receptor signaling must be taken into consideration when postulating mechanisms by which chemokines determine neutrophil responses in health and disease.

## Data Availability

The original contributions presented in this study are included in the article/Supplementary Materials. Further inquiries can be directed to the corresponding author.

## Supplementary Materials

The following supporting information can be downloaded at https://www.mdpi.com/article/10.3390/biom16010124/s1, Figure S1: Binding isotherms for CXCL8 dimer (A) and monomer (B) interactions with CS 14mer; Figure S2: Histogram plots of CXCL8 dimer binding to CS oligosaccharides; Figure S3: NMR characterization of arginine side chain interactions of CXCL8 dimer; Figure S4: Structural models of (A) CS4S-bound and (B) CS6S-bound CXCL8 dimer complexes; Figure S5: Histogram plots of CXCL8 dimer binding to chondroitin sulfate, heparin, and heparan sulfate. Videos 1 and 2 of the MD simulations of the CXCL8-CS complexes.
