# Supplementary material for: Structural Basis of Chemokine CXCL8 Monomer and Dimer Binding to Chondroitin Sulfate: Insights into Specificity and Plasticity
Source: Biomolecules. 2026 Jan 12;16(1):124. doi: 10.3390/biom16010124 (PMC12838738; doi:10.3390/biom16010124)
Supplement: Supplementary file 1 [file biomolecules-16-00124-s001.zip › biomolecules-4058853-supplementary.pdf]

Figure S1

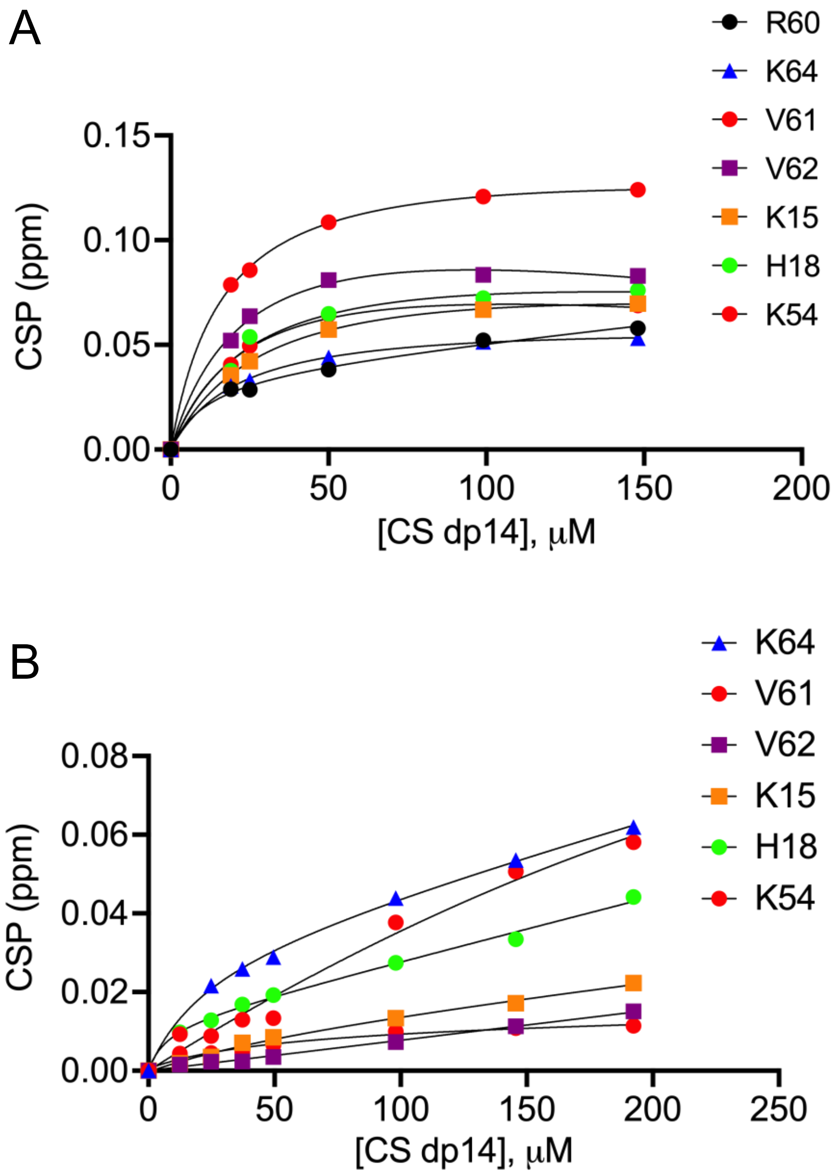

Figure S1. Binding isotherms for CXCL8 dimer (A) and monomer (B) binding to CS 14mer for a select set of residues that showed highest chemical shift changes. The binding constants were determined by fitting binding-induced NMR chemical shift changes.

Figure S2

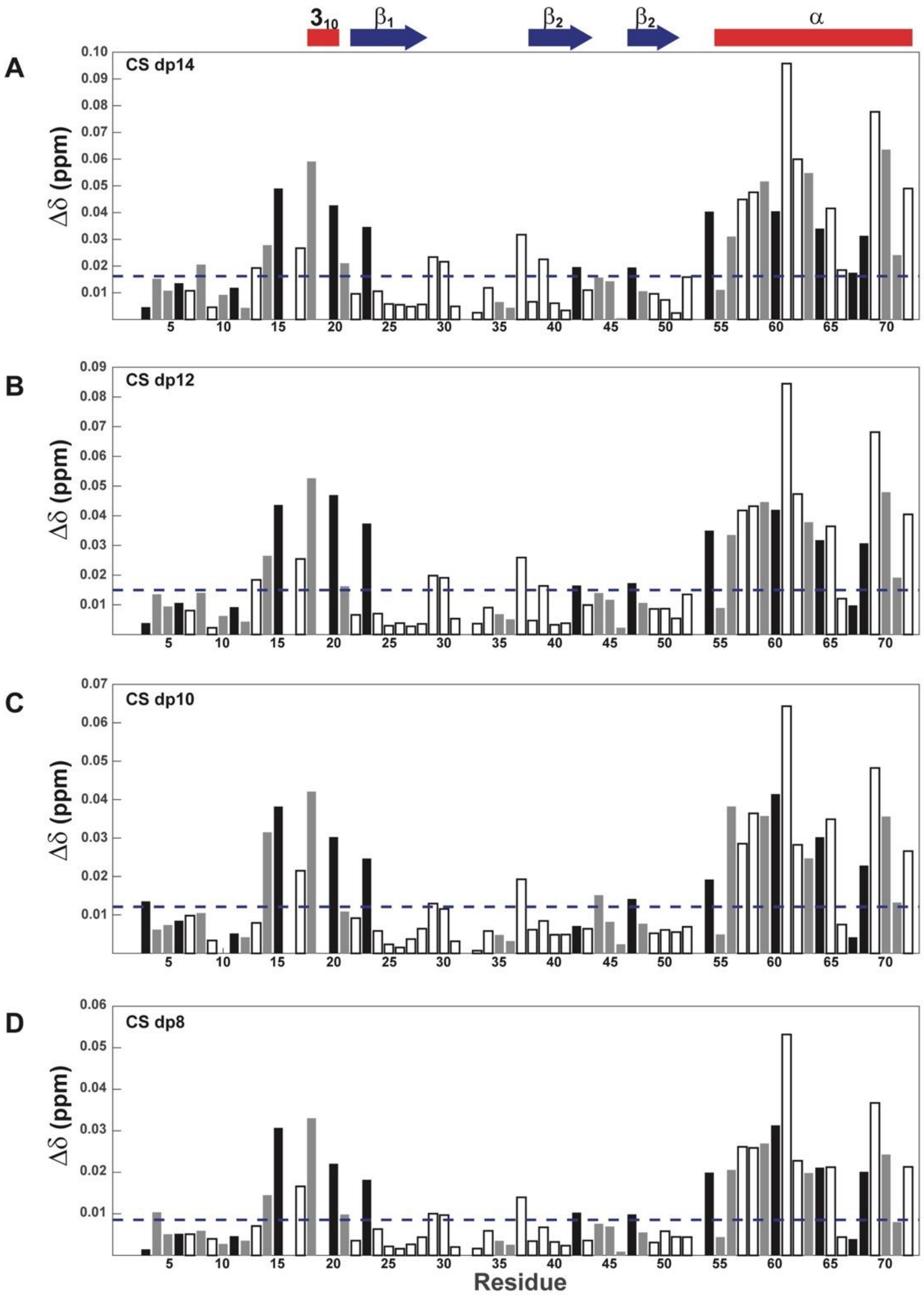

Figure S2. Histogram plots of CXCL8 WT dimer binding to CS oligosaccharides (A) dp14, (B) dp12, (C) dp10, and (D) dp 8. Basic residues are shown in black and buried residues (ASA < 40%) are shown in white. Residues showing CSP higher than the threshold (indicated by the dotted lines) are considered as involved in binding.

Figure S3

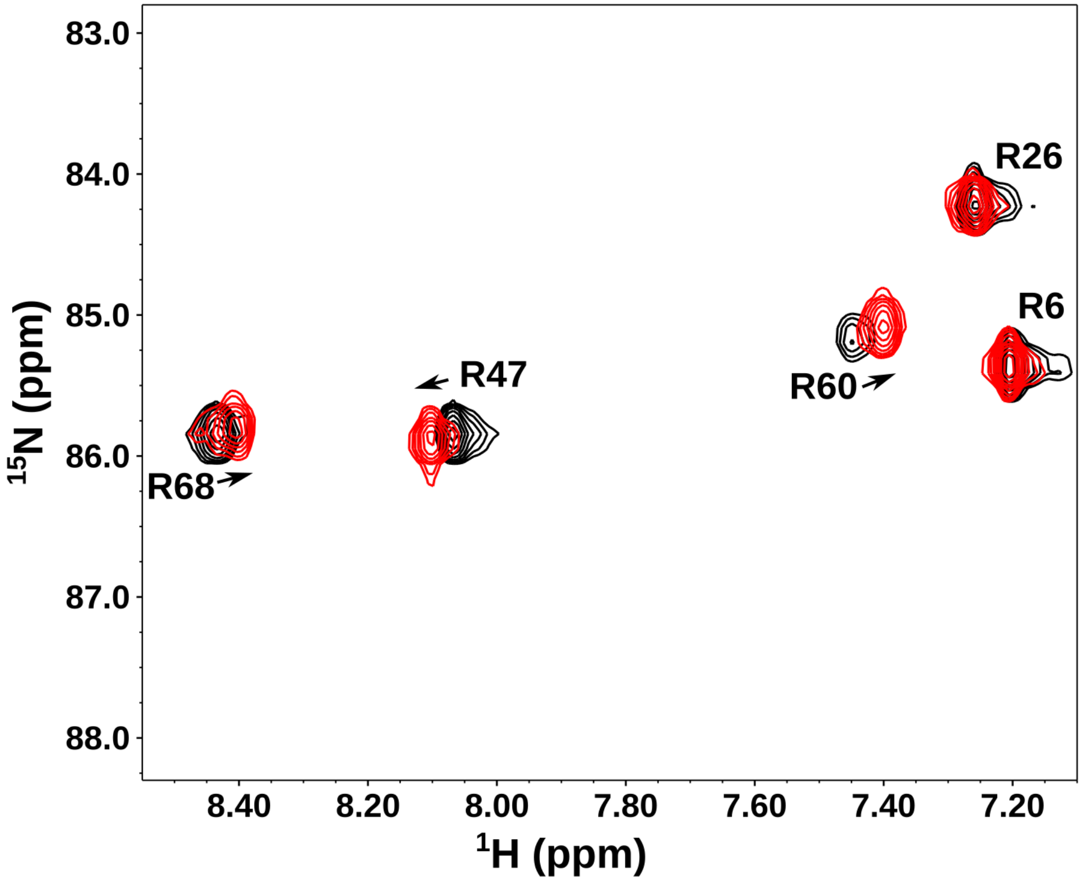

Figure S3. NMR characterization of arginine side chain interactions of CXCL8 dimer. An overlay of ^{1}H-^{15}N HSQC spectra showing the arginine side chain Nε-Hε peaks in the free (black) and CS-bound (red) forms. The spectra were collected using a 50μM sample in 50 mM phosphate pH 5.5.

Figure S4

A

B

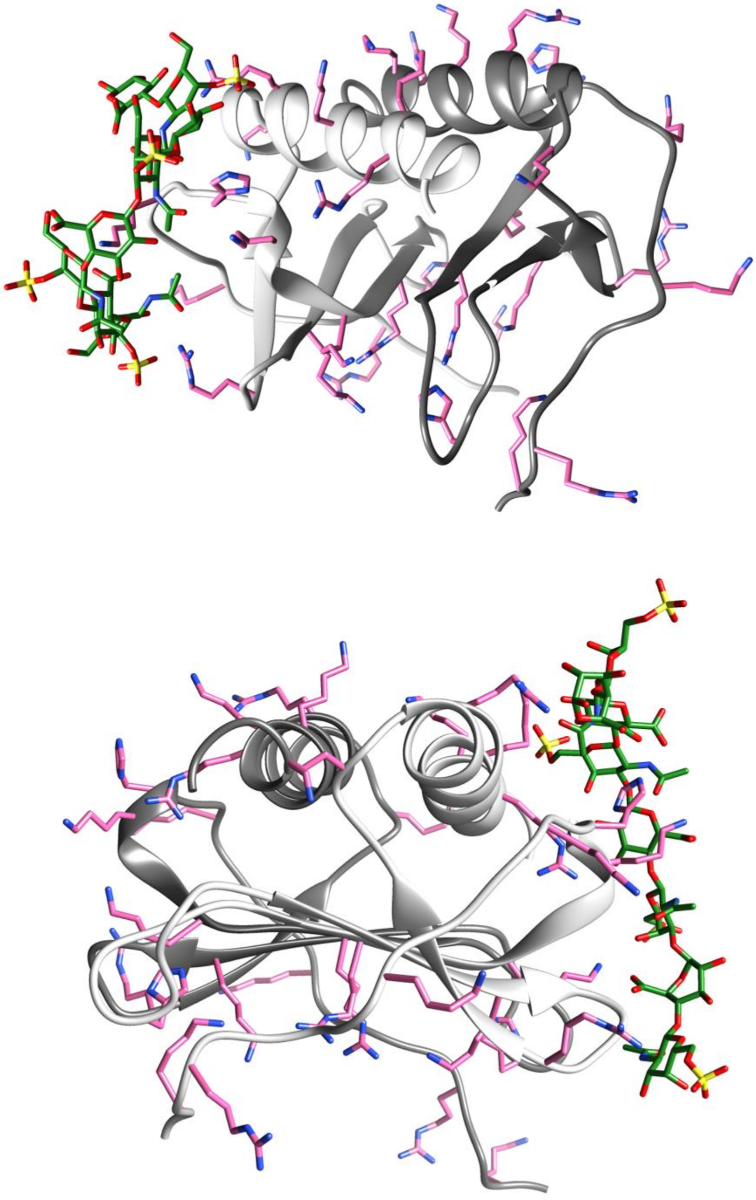

Figure S4. Structural models of (A) CS4S-bound and (B) CS6S-bound CXCL8 dimer complexes showing that binding involves interactions only within a monomer and does not involve the second monomer. The two monomers are shown in light and dark grey.

Figure S5

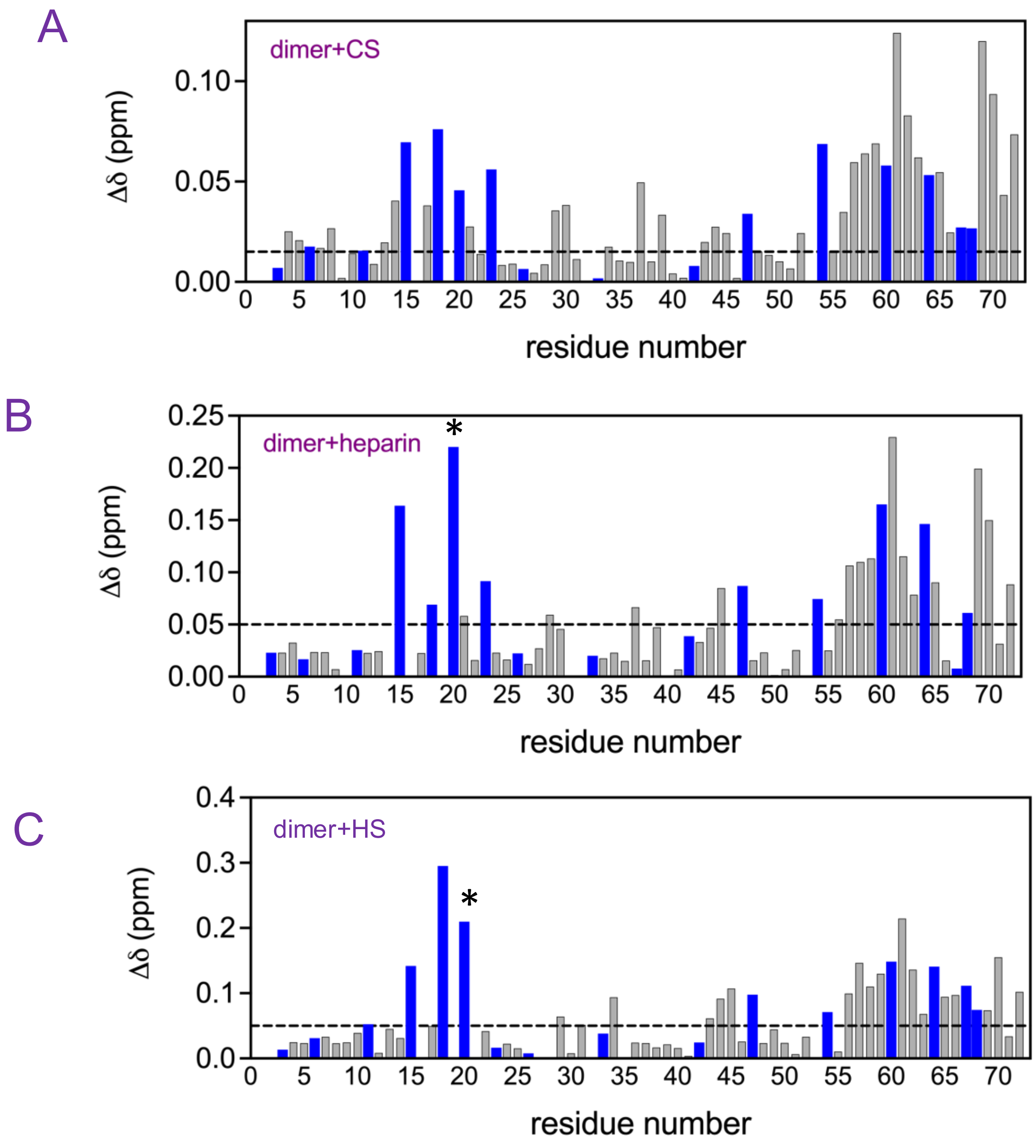

Figure S5. Histogram plots of CXCL8 dimer binding to chondroitin sulfate, heparin, and heparan sulfate. Basic residues lysine, arginine, and histidine are shown in blue. The K20 signal (*) broadens and is not detected upon binding to heparin and HS; therefore, its CSP is plotted at the same level as that observed for V61. Heparan sulfate (Catalog No: GAG-HSIII, MW ~9 kDa) was purchased from Iduron.
